# Being ready, willing and able: understanding the dynamics of family planning decision-making through community-based group discussions in the Northern Region, Ghana

**DOI:** 10.1186/s41118-020-00110-6

**Published:** 2021-01-06

**Authors:** Adriana A. E. Biney, Kalifa J. Wright, Mawuli K. Kushitor, Elizabeth F. Jackson, James F. Phillips, John Koku Awoonor-Williams, Ayaga A. Bawah

**Affiliations:** 1 Regional Institute for Population Studies (RIPS), University of Ghana, P. O. Box LG 96, Legon, Accra, Ghana; 2 Heilbrunn Department of Population and Family Health, Mailman School of Public Health, Columbia University, 722 West 168th Street, New York, NY 10032 USA; 3 Policy, Planning, Monitoring and Evaluation (PPME) Division, Ghana Health Service, Private Mail Bag, Ministries, Accra, Ghana

**Keywords:** Family planning, Community-based primary health care, Fertility decline, Rural Ghana

## Abstract

Regional contraceptive use differentials are pronounced in Ghana, with the lowest levels occurring in the Northern Region. Community-based health services, intended to promote maternal and child health and family planning use, may have failed to address this problem. This paper presents an analysis of qualitative data on community perspectives on family planning “readiness,” “willingness,” and “ability” compiled in the course of 20 focus group discussions with residents (mothers and fathers of children under five, young boys and girls, and community elders) of two communities each in two Northern Region districts that were either equipped with or lacking direct access to community health services. The study districts are localities where contraceptive use is uncommon and fertility is exceptionally high. Results suggest that direct access to community services has had no impact on contraceptive attitudes or practice. Widespread method knowledge is often offset by side-effect misperceptions. Social constraints are prominent owing to opposition from men. Findings attest to the need to improve the provision of contraceptive information and expand method choice options. Because societal acceptance and access in this patriarchal setting is critical to use, frontline worker deployment should prioritize strategies for outreach to men and community groups with prominent attention to social mobilization themes and strategies that support family planning.

## Introduction

Ghana pioneered family planning program policy in West Africa (Machiyama & Cleland, 2014). Although fertility declines have occurred since the 1970s, from 6.4 births per woman to 3.9 births per woman (Ghana Statistical Service (GSS), Ghana Health Service (GHS) & ICF, 2018), the rate of decline has currently stalled (Bongaarts, 2005, 2017). The persistence of high fertility is more evident in the Northern Region than in any other region of Ghana. The prevailing total fertility rate (TFR) is 5.8 births per woman (GSS et al., 2018). Changes in reproductive behavior occurring elsewhere in Ghana have not taken place equivalently in the Northern Region. Fertility in the region has declined only slightly, from a TFR of 7.0 births per woman in 1993 to 5.8 births in 2017 (GSS et al., 2018). While knowledge about contraceptive methods in Ghana is nearly universal (GSS et al., 2018; GSS, GHS & ICF International, 2015), unmet need for family planning is pervasive (GSS et al., 2015). Estimates indicate that 17.4% and 12.5% of married women in Ghana express survey responses that are indicative of an unmet need for spacing and limiting, respectively. The corresponding proportion of women with unmet need for spacing is highest in the Northern Region at 21.7% but lowest for limiting at 6.1% (GSS et al., 2015; Nyarko, Sparks, & Bitew, 2019). Prevailing contraceptive use prevalence remains at 31.0% in Ghana, with the substantially lower rates estimated for the Northern Region (18.5%) (GSS et al., 2018).

This paper aims to assess factors that explain the relatively low level of contraceptive usage in the Northern Region of Ghana through an analysis of qualitative data on community perceptions about family planning “readiness,” “willingness,” and “ability”. These are compiled in the course of 20 focus group discussions with community members (mothers and fathers of children under five, young boys and girls, and community leaders/elders) residing in two communities each in two Northern Region districts that were either equipped with or lacking direct access to community health services. Achieving an understanding of the social context for sustained high fertility will provide useful knowledge for deliberations on family planning operational policy development in the region. Until it was divided through a referendum in 2018, the Northern Region was the largest region in Ghana in terms of land mass with a population estimated at 2,858,793 (Ghana Statistical Service, 2016) and among the three poorest regions in the nation with the majority of residents occupied in the agricultural sector. The main ethnic group in the Region consists of Mole-Dagbanis who are predominantly Muslim and follow the patrilineal lineage systems of descent and inheritance (Ghana Statistical Service, 2013). Early marriage and polygamy are common features in this setting. Almost 40% of women (38.4%) reported that their partners had additional wives, compared to the national average of 14.0% (GSS et al., 2018). Ghana’s pluralistic legal system allows for polygyny as multiple marriages can be conducted under Islamic or customary law. However, multiple marriages under the Ordinance are illegal as this signifies bigamy which is an offence (Allott, 1958; Bankas, 1992). Gender stratification is pronounced; women have limited autonomy and authority owing to restrictive patriarchal norms. These norms are reinforced by low levels of women’s educational attainment and literacy (GSS et al., 2015). Constrained autonomy directly affects women’s health seeking behavior. For example, only 52.7% of Demographic and Health Survey respondents in the Northern Region confirmed that they could make decisions to seek personal healthcare either solely or jointly with their partners which is well below the 76.9% of women in Ghana that report the same (GSS et al., 2015). This signifies that 47.3% of married women in the Region can seek healthcare solely with their husband’s permission.

### Community-based family planning interventions in Ghana

In 1999, a national health policy initiative was instituted with a mandate to provide primary healthcare, maternal and child health, and family planning services, at the community level, nationwide. This initiative, implemented in 2000, is known as Community-based Health Planning and Services (CHPS). Its strategies were developed and tested by a quasi-experimental study of the Navrongo Health Research Centre that was conducted in the Kassena-Nankana District of the Upper East Region of Ghana (Binka, Nazzar, & Phillips, 1995). When the Navrongo Project produced initial evidence of significant fertility and mortality declines in its first 3 years of implementation, its service model was transferred to the Nkwanta District of the Volta Region (now Oti Region) where replication research was used to clarify practical means of scale-up.

CHPS was adopted as a national policy for scaling-up lessons learned from the Navrongo and Nkwanta experiments (Ghana Health Service, 1999). However, over its first decade of operation, CHPS coverage was found to be incomplete in many districts owing to a variety of resource, leadership, and strategic lapses (Krumholz, Stone, Dalaba, Phillips, & Adongo, 2015; Nyonator, Jones, Miller, Phillips, & Awoonor-Williams, 2005; Phillips, Bawah, & Binka, 2006). In response, the Ghana Health Service launched an investigation of factors that could explain poor implementation progress (Binka et al., 2009). Based on findings from this investigation, a 5-year trial was launched in 2010 to test means of accelerating CHPS implementation and reforming operations. Known as the Ghana Essential Health Interventions Programme (GEHIP), this trial of reform successfully demonstrated ways to achieve full CHPS coverage and reduce childhood mortality and fertility in the process. Expanding CHPS coverage was associated with increased contraceptive use, but pervasive unmet need for contraception was unaffected by GEHIP (Asuming, Bawah, Kanmiki, & Phillips, 2020) and fertility effects of GEHIP activities were minor (Phillips, Jackson, Bawah, Asuming, & Awoonor-Williams, 2019). Successful CHPS strengthening in the Upper East Region has improved maternal and child health without adequately addressing unmet need for family planning (Asuming et al., 2020).

In keeping with Ghana Health Service policies to test phases in program development, the GEHIP success invited the launching of a new transfer study to test the effectiveness of replicating success. Since the CHPS implementation problem was especially evident in the Northern Region which features the highest mortality and TFR and lowest contraceptive prevalence rate (CPR) in the country, study districts of this project were located in the challenging context of the Northern Region, as well as localities of the Oti and Volta regions where CHPS progress has languished.

Research guiding CHPS development has focused on social contextual factors that challenge efforts to provide community-based care. Adongo et al. (1997) provided insights into social factors that constrain family planning adoption among women in Kassena-Nankana District. This and other studies find that owing to cultural expectations about marriage and childbearing, promoting family planning use can result in social and spousal discord (Adongo et al., 1997; Bawah, Akweongo, Simmons, & Phillips, 1999). Training of workers who are responsible for CHPS mandate was based on these findings. According to policy, community health officers (CHO) were to be dispatched to communities to provide service delivery in conjunction with guidance from community leaders in the health committees and support from trained community health volunteers (Binka et al., 1995; Nyonator et al., 2005).

A series of qualitative appraisals have been conducted across Ghana assessing the influence of CHPS on family planning attitudes and fertility. These appraisals, conducted in northern (Upper East Region) and southern Ghana have consistently shown that significant improvements in contraceptive uptake could be realized if social constraints to adoption are appropriately addressed (Adongo et al., 1997, 2013; Adongo et al., 2014; Adongo et al., 2014; Dalaba et al., 2016; Nyonator et al., 2005). Recent evidence suggests, however, that these social and institutional challenges are being neglected, a fundamental oversight that is negatively affecting family planning service provision (Krumholz et al., 2015). Communities with CHPS compounds have benefits in terms of direct access to CHO oversight and services. On the other hand, communities without CHPS compounds may have access to periodic outreach services from CHOs; however, their primary sources for health services are usually health centers or hospitals in neighboring villages or towns. To address this problem, replication research, guided by GEHIP’s findings, was to be conducted in the Northern, Oti, and Volta Regions. Qualitative research can best determine why CHPS is inadequately addressing unmet need and how its services can improve the contribution of community-based care to reproductive health development (Phillips et al., 2018); hence, the need for this baseline qualitative systems appraisal.

A core strategy of this replication project, also known as the CHPS+ program, involved the creation of “Systems Learning Districts” (SLDs) where health systems strengthening interventions were constituted on the GEHIP model, with the goal of developing a platform for visiting managers and implementers of primary health care services to observe systems strengthening activities and plan their local replication effort. Our analysis assesses fertility and family planning attitudes prior to interventions and also compares the attitudes of SLD residents in communities that have functional CHPS versus communities that still lack CHPS services to determine if attitudes and access to family planning differ.

### The “Ready, Willing, and Able” framework

Community members’ perceptions about contraception are appropriately based on the “ready, willing, and able” model that has been used to explain fertility decline elsewhere (Lesthaeghe & Vanderhoeft, 2001; Machiyama & Cleland, 2014). We adopted the concepts of “readiness”—connoted by couples’ desires to limit or space births, “willingness”—an individual trait that connotes individual propensity to overcome social or spousal constraints, and “ability”—as represented by geographic, informational, or cultural access (or a lack thereof) to contraception (Machiyama & Cleland, 2014). Fertility decline is best conceptualized at the societal level since it is “grounded in culture” (van de Walle, 1992). Policy discourse has therefore focused on the notion that high fertility in rural African settings is “institutionalized” by social norms and customs that are unique to the region and best studied at the community level (Caldwell & Caldwell, 2002; Caldwell, Orubuloye, & Caldwell, 1992; McNicoll, 1975, 1980). In this context, focus group discussions (FGD) are particularly useful means for eliciting information for health systems appraisals among rural populations since the goal of such research concerns exploring community-based phenomena (Cristancho, Garces, Peters, & Mueller, 2008). The discussions allow for interactions and shared representations at a miniature societal level (Farr, 1995; Kushitor et al., 2019). Based on this framework, the main aim of the study was to identify how “ready,” “willing,” and “able” community members were to adopt family planning methods and plausible implications for policy and programmatic action that results imply. Using focus group discussion data from a qualitative systems appraisal with community members in rural settings in the Northern Region of Ghana, we posed the following research questions: (i) How “ready,” “willing,” and “able” are community members to adopt family planning methods? (ii) What are the different experiences and attitudes toward family planning and fertility across the communities with and without direct access to CHPS?

## Method

### Study design

The qualitative data were taken from a larger data corpus generated from a baseline CHPS+ qualitative systems appraisal that replicated studies conducted early in CHPS implementation to gauge community perceptions of the program and seek stakeholder advice on ways to improve its services. The entire study spans five years (August 2016 to July 2021) with baseline data collection taking place in the first year. This appraisal was conducted over the April to May 2017 period in four districts across the Northern, Oti, and Volta Regions of Ghana. These four districts were selected as SLDs for the CHPS+ program and would become “centers of excellence” in CHPS operations for their respective regions. For the larger qualitative study, eight CHPS zones comprising various rural communities in the four SLDs for the Northern (Gushiegu municipal and Kumbungu district) and Volta (Central Tongu district) and Oti (Nkwanta South municipal) Regions of Ghana were selected as the study settings. The qualitative systems appraisal sought to evaluate the functions of the primary healthcare system in the three regions prior to interventions to strengthen the health system. Thus, focus group discussions were conducted with study participants in the four SLDs. Participants for this larger study included community members across various age and gender categories, community health volunteers, frontline health workers (community health nurses, community health officers, midwives, and enrolled nurses), and district health management team members.

The interview guides used for community member group interviews consisted of information on a range of topics: community members’ health seeking behaviors, ratings of CHPS/other health facility services, ratings of service providers, challenges in healthcare provision, solutions to healthcare challenges, issues on maternal and child mortality, and issues on fertility and family planning. Community health volunteers, frontline workers, and district health management team members were also interviewed on their assessment of CHPS. However, in this paper, we limit our analyses solely to the data collected from community members in four CHPS zones in the Northern Region. Analyses are also limited to data emerging from discussions of fertility and family planning. To protect the identity of participants who contributed to the study, we term the four zones as communities A, B, C, and D, respectively. Communities A and C are functional CHPS zones in their respective districts while communities B and D are not^1^.

#### Recruitment and interview process

Community members were recruited purposively by frontline workers who have service responsibilities in selected communities and were usually aided by community health volunteers. They mobilized community interest in the study through announcements or personal visits to homes. The participants were informed of the day and time for the FGDs which took place at various locations in the communities, including at a chief’s palace, at basic schools, at CHPS compounds, and in open community spaces. At the specified interview times, community entry protocols were carried out by the field team, then eligible community members who had turned up on the day of the discussions convened for the group discussions. The plan was to engage a maximum of eight participants per group, but as indicated in Table 1, the numbers sometimes went slightly below or above this. The community focus groups were formed to discuss a range of topics related to healthcare access, health seeking behavior, and perceptions about CHPS in addition to family planning and fertility which are the focus of this paper. The team encountered no obstacles regarding topics discussed in each meeting. Further details on recruitment during the CHPS+ baseline qualitative systems appraisals are indicated in other studies (Kushitor et al., 2019; Wright et al., 2020).

**Table 1:** Number of participants distributed across the five groups by community

Participant type:	Communities of Kumbungu district		Communities of Gushiegu district	
	Community A (functioning CHPS)	Community B (non-functioning CHPS)	Community C (functioning CHPS)	Community D (non-functioning CHPS)
**Mothers**	8	8	8	8
**Fathers**	8	8	8	8
**Young girls**	8	8	6	8
**Young boys**	8	8	8	8
**Leaders/elders**	8	11	7	10
**Total**	**40**	**43**	**37**	**42**

A study team of ten, consisting of one supervisor, six male interviewers, and three female interviewers, travelled to the various communities and facilitated the conduct of FGDs. For each community, all six FGDs were conducted in a single day so that group discussions spanned four days. To ensure rigor in the data collection process, various features in the study design related to interviewer selection were taken into consideration. The nine interviewers had some form of tertiary education and were proficient in Dagbani, the local language that all group interviews were conducted in. In addition, interviewers had knowledge of customs in these settings, and they all had experience conducting focus group discussions. Males interviewed young boys, fathers, and elders while females interviewed mothers and young girls. For this community-based study, these interviewer characteristics were important to invoke community trust. Employing same-sex interviewers was especially important in ensuring participants were comfortable enough to share societal views on the sensitive subject of reproduction. The interview guide used for the FGDs was semi-structured, enabling a systematic approach to questioning while allowing for probes to obtain a depth and range of responses. To ensure the validity of the instrument, the guides were assembled by experts in the field and were then screened and refined prior to use. The group discussions were held at various locations with venues selected in consultation with frontline workers. Session durations ranged from 50 to 140 mins.

#### Study participants

Focus group discussions were conducted with five groups of community members in each of the four communities: (1) mothers of children under age five, (2) fathers of children under age five, (3) male youth without children (ages 15 to 24 years), (4) female youth without children (aged 15 to 24 years), and (5) community leaders/elders (see Table 1 for a list of the groups with numbers of participants).

The majority of participants in all four communities were Muslim. While mothers and community leaders/elders had no formal education, all youth had some form of secondary education, and fathers had a mix of none, junior, and senior secondary education. The average ages of mothers, fathers, young girls, young boys, and elders/community leaders in Kumbungu were 33 years, 37 years, 17 years, 20 years, and 56 years, respectively; while in Gushiegu, average ages of group discussion participants were 32 years, 30 years, 17 years, 18 years, and 58 years, respectively. Furthermore, there was an average parity of 4, 5, and 8 children among Kumbungu mothers, fathers and elders, respectively, and a respective average of 4, 4, and 10 children among mothers, fathers, and elders in Gushiegu.

#### Data analysis

The group discussions were recorded and later translated, transcribed, and back translated for accuracy. The four transcribers were participants in the field team that conducted the group discussions. Using thematic analysis (Braun & Clarke, 2006), the transcripts were analyzed aided by the qualitative data analysis software, Atlas.ti versions 7.5.1 and 7.5.18. Three researchers (three of the authors) jointly generated codes for a sample of the transcripts and through this developed a coding frame which was used to guide the coding of subsequent transcripts, and thus established inter-coder reliability. The entirety of the transcripts were coded, bearing in mind the research questions, while also paying attention to inductive codes that would emerge. Codes generated on family planning and fertility topics were examined and grouped into their peculiar themes (Kushitor et al., 2019; Wright et al., 2020). To gauge the possible impact of CHPS exposure on family planning discussion, analyses were further conducted by observing patterns and comparing responses between communities with and without functioning CHPS compounds. Responses that were unique to communities either with or without CHPS were noted as differences between them.

#### Ethical considerations

The study protocol was reviewed by the Ghana Health Service Ethical Review Committee, Accra, and by the Columbia University Institutional Review Board in January 2017. Written informed consent was obtained from literate study participants with oral parental consent obtained for all girls and boys who were under 18 years of age. Participants who were illiterate were asked to thumbprint the consent forms to indicate their consent. Refreshments and soap were provided to all study participants as reciprocity.

## Findings

The findings are discussed based on the categories of participants’ “readiness” to space or limit fertility, “willingness” to adopt contraceptive methods amidst social and spousal constraints, and “ability” to access contraception through sources of information and services, and perceptions of services.

### Readiness

The notion of readiness signifies one’s desire to reduce fertility. Responses evidenced the pronatalist (or pro-birth) stance of the participants. They discussed their desires to space or limit births while also offering the reasons why they wanted to have many children. The two main themes discussed were (1) the desire to limit and space children and (2) the value of children. An assessment of responses also indicated no clear differences in “readiness” between participants with and without functioning CHPS compounds.

#### Desire to limit and space children

Mothers consistently reported desiring fewer children than they believed that their husbands/partners desired. Young participants reported wanting fewer children than older participants, reflecting the view that having few children fosters their education and supports prospects for a healthy and prosperous life. Yet, gender discordance was commonplace:

Participant 1: Left to the women alone, we will want to have five children at most, but the men would not agree.

Participant 2: The men want 12 children (Community A mothers, CHPS functioning)

Similarly, in a community that lacked CHPS,

Interviewer: What about the men, what is the ideal number of children?

Participant 2: For the men, if they get 100, they will like it.

Participant 8: Unless you the woman refuses, he would not stop. (Community B mothers, CHPS not functioning)

Relative to men, mothers and young people emphasized their role as caregivers of children. Mothers and young girls typically complained that men failed to take appropriate responsibilities for their children, such as paying school fees and hospital bills, while expecting to receive the respect and prestige accrued from having many children. However, there was one view, grounded in religious tenets, that provided alternative insights on men’s childrearing responsibilities:

The idea of family planning is good, because our religion frowns on having children that you cannot cater for. So, in general, it is a good thing to do. (Community A fathers, CHPS functioning)

Fathers and elders also discussed the desire for many children and their expectation to marry many wives who could bear them several children.

For me I want to have forty children, because I have four wives and I want each to bear me ten children. (Community A fathers, CHPS functioning)

Some participants noted the fundamental difference between limiting and spacing, where spacing was perceived to be necessary for sustaining the health of mothers and children, while limiting was typically opposed due to the desire for many children. A participant from a community leader FGD stated:

We were not told the truth about family planning, the only reason we refused to accept it was the name they gave to it, reducing of child birth, how can you tell me the number of children I should give birth to. That was the reason why some people didn’t accept it. We were told to give birth to either 3 or 4 or 5 and that was what they used to spoil the name of family planning not knowing they were trying to tell us to space our children for them to be stronger. We want our children to be many, we want our household to be full of people. (Community A elders, CHPS functioning)

#### The value of children

A major reason for wanting many children stemmed from the perception that children are indicative of wealth, assets, and labor. Children perpetuate the lineage and engender community respect for their parents. Their prominent occupations as farmers and traders were also a major factor in needing more hands to work. Parents could anticipate that their children would eventually care for them, with the expectation that their homes and sources of livelihoods would be taken up by their offspring and that this intergenerational support would ensure that they would be cared for.

The perceived social benefits of childbearing were amplified by gender stratification norms. As one young girl noted,

….the men adore many children because it is an added advantage when you are contesting for a chieftaincy title. It is assumed that you are rich and responsible enough to lead people. (Community B young girls, CHPS not functioning)

Having many children is also believed to represent a form of social insurance. Since children may die, go mad, become deviants, or fail to support aging parents, having more children increases chances that some will appropriately support their parents as they age. As participants stated:

They say if you have many children, there is a high chance of them prospering. (Community D mothers, CHPS not functioning)

Infant and child mortality rates have always been high in this context. Under-five mortality is currently 77 deaths per 1000 live births in the region (GSS et al., 2018). This has consequences for reproductive motives.

I would like to give birth to 20 children…Because if you don’t give birth to many and some die, you would be left with nothing. (Community A elders, CHPS functioning)

Childlessness was considered “a heavy load to every human being”. This community B mother also mentioned that, “If you do not have a child then you have no respect. A woman is insulted by the rival because she is childless”. Thus, in this polygamous context, childlessness—whether through infertility or mortality—has only negative implications.

Overall, this readiness component highlights gender and age differentials in relation to the desire to limit. Although children are valued by all, for various reasons, there was more of a readiness from women and youth to limit childbearing while fathers and elders preferred to space children.

### Willingness

“Willingness” refers to community members’ perceptions that constraints to family planning should be overcome and contraception should be practiced despite social barriers. Attitudes to family planning were mixed as discussions resulted in both favorable and negative stances. A discussion of traditional methods also featured in almost all group discussions across districts and this provided a deeper understanding on their thoughts about abstinence. The main themes discussed include positive attitudes toward and barriers to adopting family planning methods, knowledge about family planning, and abstinence as a traditional method of spacing births. Again, comparing responses by participants in functional and non-functional CHPS communities, there were no differences on the perceived willingness of community members to use contraception.

#### Positive attitudes toward family planning methods

Generally, attitudes about family planning were positive as the idea of it was considered “a good thing”. Community leaders mentioned:

…we haven’t seen anyone in the community who says he doesn’t accept it, nobody has never said he or she does not like the family planning. Everybody has accepted it in this community. We cannot say everybody is practicing it but accepting it everyone has accepted it. (Community A elders, CHPS functioning)

But family planning is generally a good thing. Everybody in this community has endorsed it. (Community D elders, CHPS not functioning)

All group discussions across the four communities discussed positives about family planning. These broadly centered on it promoting health, alleviating/preventing poverty, preventing teenage pregnancy, and promoting fidelity in marriage. Some of these positive attitudes contradict statements indicated earlier from members of the same group that concerned non-acceptance of family planning in their community. However, such discordance is expected to generate from focus group discussions as participants may offer contrasting representations of a phenomenon.

Most groups mentioned that family planning aided in spacing births which ultimately produced healthier families, with reduced risk of maternal and child mortality and healthier children and mothers. One participant in community C noted:

People believe family planning is a good thing because it helps them space their children and also helps them take good care of their children with regards to school fees, hospital bills and what have you. (Community C young boys, CHPS functioning)

Family planning use also prevented poverty as reduction in pregnancy frequency allowed women to work. In addition, the children were an adequate number for parents to provide for.

Participant 1: Family planning is a good thing because it prevents poverty….

Participant 2:… both men and women practice it, and they believe it reduces poverty within their families. (Community D fathers, CHPS not functioning)

Among young people, family planning was perceived to be a means of preventing teenage pregnancy. Community C young boys, community A young girls, and community D fathers highlighted its use among young people to prevent early and unintended pregnancies.

Use of family planning also suggested to some men that they could have sex with their wives soon after childbirth, a perception that was associated with the notion that this prevented men from engaging in extra marital affairs. Some male participants believed that family planning was associated with healthier marriages by reducing chances of the transmission of sexually transmitted infections.

… it is a good thing. Some of us prefer to always have sex with our wives to avoid extra marital affairs, so using a family planning method is good for us. (Community D fathers, CHPS not functioning)

… it helps us the men to be faithful, and prevents us from contracting diseases. (Community C fathers, CHPS functioning)

These findings suggest ways family planning can positively reinforce marital relations through regular sexual intercourse with one’s spouse.

#### Knowledge about family planning methods

Although knowledge of at least one contraceptive method was virtually universal, considerable variance was evident in methods spontaneously cited by each group (Table 2). Community residents with and without CHPS compounds cited specific methods, namely the condom, pill, implant, and injection. Injections were the most mentioned method, stated by 16 out of the 20 groups. Two groups even gave specific additional procedural information, such as this:

…some people use the injections. We have an injection for a month, two or three, it all depends on your preference. (Community B fathers, CHPS not functioning)

**Table 2:** Family planning methods discussed by focus group discussion participants

Contraceptive methods mentioned by one or more participant^**a**^	Participant types				
	Mothers^d^	Fathers	Young girls	Young boys	Elders
Injection	**++-+**	**-+++**	**-++-**	**++++**	**++++**
Oral contraceptives^b^	**+--+**	**+-+-**	**--++**	**+-+-**	**-++-**
Emergency contraception	**----**	**-+--**	**----**	**----**	**----**
Implant	**+--+**	**+--+**	**----**	**--++**	**---+**
Condom	**+---**	**++++**	**-+--**	**++++**	**-+++**
Vasectomy	**----**	**----**	**----**	**----**	**---+**
Tubectomy	**----**	**----**	**----**	**----**	**----**
Periodic abstinence^c^	**----**	**----**	**++--**	**-+--**	**----**
Withdrawal	**----**	**---+**	**+---**	**----**	**----**

^a^Cell entries “+” indicate that the method was mentioned while “-” indicates that the method was not mentioned by group participants in the community A and community B communities of Kumbungu District and community C and community D communities of Gushiegu District, respectively

^b^Pills that the user inserts or swallows

^c^“Periodic abstinence” refers to the rhythm/calendar method. However, abstinence for a long period (2 to 4 years) until the child is older was also mentioned as a method

^d^Community C mothers mentioned medicines and family planning in general; they did not mention specific methods. In all sessions, no direct attempts were made to probe for knowledge of specific methods

Condoms were mentioned 13 times, by all fathers and young boys as well as three out of the four elders’ groups. The only two female groups to discuss condoms were one mothers’ and one young girls’ groups, both in Kumbungu district. One unusual reference to condoms was expressed by the community B young girls’ group:

We were taught in school that condom use is one of the ways to prevent pregnancy. We however get pregnant even using a condom because some of our boyfriends intentionally perforate a hole into the condom and deny being responsible when you get pregnant. (Community B young girls, CHPS not functioning)

The pill was mentioned by 11 groups, with variations. One group cited both oral and vaginal forms of the pill while another group referred to the emergency contraceptive pill. Two young girl and one young boy groups discussed learning about the menstrual cycle and a girl’s safe period as a means of preventing pregnancy. Permanent methods were rarely discussed; the vasectomy was mentioned by an elders’ group while the tubectomy was never discussed. Withdrawal was also rarely mentioned. Young girls stated the following:

Sometimes in science [class] they teach us the safe period a woman can have sex. They also taught us the withdrawal method. [Laughter] That is all. (Community A young girls, CHPS functioning)

Finally, although induced abortion is not a contraceptive method, women in a particular community may be using it as an alternative to birth control. During a young girls’ group discussion, one participant made reference to women’s use of induced abortion as a substitute for contraception. This was the only mention of abortion from the discussions.

I do not think the people of this community know of any method to avoid pregnancies because they are always seen in the hospital seeking help to abort a pregnancy. (Community B young girls, CHPS not functioning)

#### Barriers to family planning method adoption

Most participants expressed at least some superficial knowledge about family planning, while also stating a perceived need for more information from professionals about contraception. This lack of detailed information was a barrier to contraception use, promoting misconceptions and associated fear of side effects.

Although spousal relations were commonly cited barriers to adoption, concerns about side effects were mentioned in every group discussion, making this the most consistently cited reason for non-use of modern contraception. Such concerns were expressed as worries about infertility, weight gain, future complications associated with conceiving and childbirth, early onset of menopause, propensity for contracting illnesses, and concerns that family planning could cause death. Women offered these negative community perceptions they had learned:

They also think you will die or will never give birth again when you use it [family planning]... (Community B young girls, CHPS not functioning)

… They say it [family planning] gives sickness. (Community C mothers, CHPS functioning)

Of these concerns, the most common was the view that contraception induces permanent infertility, a side-effect that could jeopardize marital stability.

What this man said is the reason why people do not want to practice it because if you do it and later cannot conceive, then you are in trouble. That is the one of the reasons scaring people away from the family planning method. (Community A fathers, CHPS functioning)

CHPS and non-CHPS communities alike discussed ignorance borne by either not getting the needed information or refusing to know about family planning. Both young girls’ groups in Kumbungu mentioned being bypassed by health personnel who only discussed family planning with their mothers. In addition, doctors were not informing them about the side effects associated with the methods, while some believed health workers were deceiving them to deter them from having many children. More about informational access to family planning or a lack thereof are discussed in further detail in the ensuing section.

Another reason for attitudinal resistance stemmed from the desire for many children. People wanted many children, and family planning methods not only prevented pregnancy, there was a potential to complicate the process. One participant stated:

Some of the men want many children so they get angry when their wives use any family planning method. (Community A young girls, CHPS functioning)

Religious and traditional reasons were discussed by the following five groups, community C elders, community B young girls, young boys, and fathers, and community D young boys, who mentioned these reasons for community members’ opposition to contraception. Participants believed that in their context, the norm was to avoid family planning since their grandparents, parents, and elders had never used these methods. A young girl in community B noted that “…for the people of this community, especially the men, they say that a real Dagomba is above family planning”; thus, highlighting some of the dominant thoughts of Dagomba men on contraception.

Without providing specific details, community B young boys and girls simply stated that some religious doctrines did not allow use of contraception. Community B is a majority Muslim community, and these young people believe that certain tenets in their religion’s doctrine result in barriers to contraceptive use. Fathers in the same community stated more specifically that some Islamic scholars advise that they not use contraception. This finding provides additional insights on some religious leaders’ views on family planning. Although societal norms and religion were not major factors explicitly mentioned in all group discussions as reasons for non-use of contraception, they play a role in how women rationalize their decisions to continue bearing children despite wanting to stop. A man’s ability to marry multiple women and to divorce women for various reasons could determine whether women exert their sexual and reproductive autonomy or not.

Stigma associated with family planning attributed to prospects that contraception could foster promiscuity among unmarried young girls or infidelity among married women. One young girl stated:

Family planning is perceived as a bad idea especially for us the adolescents; because we are considered as naughty or bad when we do it. (Community B young girls, CHPS not functioning)

The ability to prevent pregnancy provides an avenue for young girls and married women to engage in extramartial sexual relationships without getting pregnant which is the main way women can get caught. Spousal mistrust has been noted in recurrent anthropological research on the gender stratification effects of polygyny (Adongo et al., 1997; Adongo, Phillips, & Baynes, 2014). However, discussions went beyond accusations of infidelity when women used contraception to address her perceived inability to conceive, which is also a major disruption to a marriage. Concerning this, men’s and women’s groups stated:

Many people are divorced because of that family planning thing. The woman probably did it and later wanted to conceive but could not. (Community B fathers, CHPS not functioning)

For the men they do not care because whether you use it or not it does not affect them. If you use it and experience complications, they can always marry a different woman. (Community D mothers, CHPS not functioning)

Other participants also discussed people’s secret use of contraception which was linked to shyness, fear of angry husbands, and disapproval of community members. Financial inaccessibility to contraception was only mentioned as a barrier to use in conjunction with women’s inability to pay for it and needing their husband’s permission and financial assistance. Discussions about men’s influence regarding contraceptive use featured infrequently. References were made to women needing consent from husbands to use a method. Consenting husbands would approve of women’s use and then it becomes her responsibility to make sure it is used appropriately. On the other hand, disapproving/indifferent husbands would lead to non-use or marital disruptions once complications emerged from her use.

#### Traditional methods for spacing children

Some groups noted that community members spaced children naturally through abstinence. Opinions about this practice were mixed, however. Some discussion groups viewed abstinence as an effective means of spacing births while others highlighted the detrimental family effects of women leaving their husbands and families to reside in their parental household for several months. And women who abandon such customs and get pregnant soon after childbirth are stigmatized. The health rationale for spacing was generally acknowledged, while close birth spacing was derisively viewed as being tantamount to loving sex.

Participant 7: [Sighs!] When you deliver, it is like your husband has also delivered. If you are seen in your husband’s bedroom, you are gossiped about; people say you love too much sex, how can you have a small baby and want your husband to make love to you, is what people would ask.

Participant 2: Whenever we deliver, our husbands also deliver because there will be no sex until the child is up to two years and two months. At that age, the child is old enough for you to conceive again, if you mistakenly get pregnant before your child gets to that age, nobody would eat when you cook in the compound.

Interviewer: Why?

Participant 2: They will say you are filled with filth [Laughter]. (Community B mothers, CHPS not functioning)

One father acknowledged their use of abstinence to prevent births:

We practiced family planning for a very long time without the white man’s idea of family planning. We abstain from sex anytime our wife has a baby, we do not get close to her not to talk of touching her. (Community A fathers, CHPS functioning)

This traditional method can also be a means of asserting agency. Women in community B mother’s group discussed their strategy of using abstinence as a means of punishing their husbands by making them wait.

Participant 2: …They are too difficult and that is why we make them wait up to 2 years. For me, if a man does not make me happy, he would not get to make love to me.

All others: [laughter]

Participant 2: Yes, even if I do family planning I will still deny him. (Community B mothers, CHPS not functioning)

In other mother’s discussion groups, participants mentioned the frequent failure of men to adequately provide for children, in the context of justifying denial of sexual relations. As an extension of this perspective, one mother expressed the view that substituting contraception for abstinence would tend to subject her to pressure for unwanted sexual relations:

But that is what our mothers used to do. When they give birth they stay away from sex for 3 to 4 years. Now that they have brought these methods, it means that even after delivering, you do not rest, you have to continue with the sex and that is not fair. (Community A mothers, CHPS functioning)

This line of discussion suggests that a pregnancy soon after childbirth was a factor driving denigration, not necessarily sexual relations soon after delivery. Thus, women using family planning soon after delivery felt resigned to resume sex with their partners, whether they wanted to or not. These women’s attitudes seem to convey that they derive some level of agency or sexual autonomy from this tradition of abstinence that family planning may take away.

The findings indicate a willingness of community members to use contraception but a reluctance to implement preferences owing to social and spousal costs of fertility regulation and perceived risk of side effects. In addition, participants highlight the fact that the concept of family planning was not new, as pre-Western contraception involved years of abstinence post-childbirth.

### Ability

The “ability” of participants was reflected in their access to physical/geographical and informational family planning services as well as perceptions about these services. While earlier findings revealed no distinct difference in terms of family planning knowledge among participants from functioning and non-functioning CHPS communities, as expected, their sources of knowledge did differ.

#### Sources of family planning information and geographic access to services

Community members mentioned a variety of sources of family planning information and services. Participants in communities with functioning CHPS received services from health staff at CHPS facilities while those without CHPS had access through health workers at hospitals or health centers. Community members in communities without CHPS mentioned utilizing the following health facilities: community B—Kings Medical Center or Bontanga Health Center and community D—Kpatinga Hospital, Gushiegu Hospital, or Nalerigu Hospital. All communities also had services provided by volunteers.

The main modes health personnel provided contraception information and services were through clients visiting the CHPS compound or the hospital, through counseling at outreach clinics and during group meetings and home visits with community members. One mother’s group noted the frequency and means through which their health workers talked to them about family planning.

Everyone in this community has accepted it because the health workers speak to *us all the time about it*. Even recently, we were gathered and spoken to about family planning. They also talk to us about it when we visit the hospital [CHPS compound]. (Community A mothers, CHPS functioning)—italicized for emphasis

Generally, group discussions entailed participants travelling to either the CHPS facility or another health facility to get family planning information and services. Groups mentioned the following:

If they do not want to get pregnant, they go to the CHPS compound for advice and the necessary things are done. (Community A young boys, CHPS functioning)

…they always talk about it at the Kumbungu Hospital. Some of us feel shy to do it. I have witnessed some people doing it at the Kumbungu Hospital several times. (Community B fathers, CHPS not functioning)

Another means of family planning discussions with women was through scheduled outreach clinics at the health facility:

The nurses speak about family planning when the women send their kids for weighing or immunization. (Community C young girls, CHPS functioning)

The strategic role of CHOs providing family planning information and supplies through home visitation was discussed, albeit infrequently, despite its usefulness in eliminating the need for men and women to travel to a CHPS compound before receiving services. One father acknowledged the role of nurses in providing education during home visits:

Mostly the nurses go from house to house to educate us on these methods. (Community A fathers, CHPS functioning)

Organized community meetings and gatherings were also opportunities to provide detailed family planning information and opportunities to adopt a method:

It is from the nurses, they started talking about it to us. The nurses at the facility. They have seen that family planning is still an issue in the community so any gathering we have they talk about it to us. (Community A elders, CHPS functioning)

Last three weeks we had a meeting with the family planning experts, they separated we the men from the women, they educated us on the methods for males and females, so you choose the one that suits you. (Community A fathers, CHPS functioning)

The health personnel that attended to community members were mostly nurses followed by volunteers and doctors. The fact that nurses were the most cited means of acquiring information about family planning highlights their importance in this context. Participants from functional and non-functional CHPS compounds alike mentioned volunteers providing services. A father stated the following:

Sometime ago, the volunteers gave me these family planning contraceptives to share to people, initially, they did not want to come for it, but when they realized it was a good thing, people were rushing to my house for it. (Community B father, CHPS not functioning)

Some community members had also been trained at Kumbungu Hospital to discuss family planning issues with other women. Schools, the media (radio and television), and NGOs were other sources of information. Young boys in community B also mentioned their peers as informal sources of family planning information, in addition to more the formalized peer education at school.

Despite the range of sources and information, barriers to contraception existed, due to a lack of detailed information leading to sparse knowledge. Participants also felt some sources of information were more reliable than others. One mother stated:

We do not want to use it [family planning] because the doctors themselves are not talking to us about it and so we are afraid. We really do not know how it works and until they come here to educate us more on it we will continue to be afraid. (Community D mothers, CHPS not functioning)

As mentioned earlier, one particular group was overlooked by health workers—young girls in the Kumbungu District. They reported being bypassed by these health workers, thereby limiting their access to information from this preferred source but reported receiving information from community groups and clubs.

This final component of ability to access family planning services indicates varied sources for communities with and without CHPS compounds. Health personnel and health facilities were primary modes of information and methods.

## Discussion

The findings gathered from group discussions in these four communities in the Northern Region reiterate themes on awareness and perceptions about family planning as well as socio-cultural barriers to contraceptive use, from discussions compiled almost two decades ago in the course of focus group sessions with community members in the Upper East Region (Adongo et al., 1997; Bawah et al., 1999), as well as more recent studies across parts of rural Ghana (Doctor, Phillips, & Sakeah, 2009; Nettey et al., 2015), and among marginalized groups (Mprah, Anafi, & Yeaboah, 2017). However, aspects of our current results suggest that normative change in attitudes may have occurred, as suggested by other recent qualitative assessments (Adongo et al., 2013; Dalaba et al., 2016). The Northern Region has the highest fertility level in the country, and this change is needed in order to achieve declines in fertility. This evidence of improved social support for family planning could translate into further use depending on a variety of factors. Basic information about family planning methods and points of care is now widespread. Evidence from this study is consistent with the proposition that the initial scale up of the CHPS initiative to the Northern Region, in addition to the hospitals, has improved the provision of accessible family planning services to rural poor pronatalist settings in the districts studied. The Ministry of Health relies on donor support for family planning commodities supplied to all levels of healthcare. These efforts ensure, to an extent, accessibility to needed commodities even at the primary healthcare level. The provision of community health personnel that are available and ready to provide services has also facilitated community knowledge of family planning. Unfortunately, although availability of services through fixed health facilities may have fostered knowledge, it has not had the intended impact on attitudes, preferences, or use.

In relation to willingness to use contraception, fear of side effects was the most commonly cited reason for current and future non-use. A range of methods exist; however, experiences of side effects result in discontinuation, as noted in other studies (Adongo et al., 2013; Adongo, Tabong, et al., 2014; Dalaba et al., 2016), and these concerns cannot be dismissed. Despite the issue of side effects and other negative attitudes producing barriers, family planning was considered as generally favorable in all the communities. Results show that in this context contraception is best framed as a method for improving maternal and child health than for limiting births. This indicates that although births may be mistimed they are rarely unwanted. Bawah, Asuming, Debpuur, and Phillips (2016) found that births whether declared wanted or not yielded similar health outcomes, highlighting the pronatalist nature of that setting which could hold true for this as well.

In terms of readiness to limit childbearing, there is a still a strong desire for children by all groups and a heavy stigma associated with childlessness. Community members value children as they are a resource that guarantees their wealth in many ways—children ensure their respect/prestige in the community, security in old age, labor, and more—and this transcends financial wealth. The desired numbers were higher for men and elders’ groups and lower for young people and mothers’ groups. This suggests a readiness to limit among those who tend to bear the cost of childbearing and childrearing (women, young girls, and sometimes young boys) as they were the ones that opted for fewer desired numbers. However, as long as culture leans toward pronatalist tendencies, continuous advancements through tailoring family planning outreach to fit community needs are still necessary. For example, health personnel referring to contraception as a means to space and not limit childbearing resulted in improved attitudes, and such culturally sensitive advancements can ensure increased uptake.

The findings indicate no clear differences between communities with or without CHPS compounds with respect to readiness and willingness, but sources of family planning information that led to ability to use contraception did differ. Dalaba et al. (2016) found similar themes between community members exposed to “only CHPS” and “both the Navrongo Project and CHPS”. CHPS is currently functioning as a clinic-based program rather than a system of care that is truly community-based (Odugleh-Koleva & Parrish-Sprowl, 2018). For CHPS to be socially engaged and community present, its services must reach out to groups of men, utilizing means of communication that men routinely pursue. The willingness of women to practice family planning is evident, but their reluctance to confront husbands with their preferences is evident. For the program to succeed, social access must be pursued. By this, we mean the importance of augmenting clinical care with community outreach that addresses social constraints to family planning (Odugleh-Koleva & Parrish-Sprowl, 2018). In the absence of active social engagement, convenient CHPS clinical care will have no impact. Exposure, regardless of CHPS in the community, highlights the importance of other sources—hospitals, health facilities, schools, media—in promoting family planning awareness and influencing attitudes. Social interaction and a diffusion of family planning information may be additional mediums of knowledge as well as reasons for all the misconceptions men and women may have (Rutenberg & Watkins, 1997). It was also surprising that the presence of CHPS in a community did not result in less negative attitudes. Quantitative studies can further determine significant differences, especially with actual contraceptive use, based on CHPS exposure.

Finally, it was acknowledged that reproductive decision-making and use of family planning is conferred on men. In this patrilineal society, it is understandable that bridewealth payment “secures the woman’s womb” and so men gain their sexual and reproductive autonomy in this regard (Horne, Dodoo, & Dodoo, 2013). Women’s use of contraception sometimes leads to them being stigmatized as immoral or being divorced when found to be barren through its use. These negative impacts from contraceptive use may have socio-economic and other social implications for women. This finding relates with the theme of males as gate-keepers, and this can ensure positive results if harnessed correctly (Adongo et al., 2013; Ngom, Debpuur, Akweongo, Adongo, & Binka, 2003). Females are generally disempowered with regards to fertility and family planning decision-making in such patriarchal settings, although some women expressed their agentic views on the subject. The complex nature of these issues highlights the need to understand gender relations in cultural contexts as a major strategy to promote contraceptive use. Furthermore, although men in this patrilineal setting were supposed to provide for their children belonging to their lineage, women complained that they were not doing so. This was similar to findings from a study conducted in a matrilineal setting (Adongo et al., 2013).

## Conclusions

Findings from this investigation have implications for program action. First, discussions indicate a degree of support for family planning that would be consistent with intensifying the promotion of services if care is taken to ensure that themes and activities are culturally appropriate.

Second, there is no evidence that geographic access to CHPS service points has had an impact on perceptions about childbearing or the practice of family planning. Findings suggest that passive service point provision of care has limited behavioral effects in this societal context. Clearly, the CHPS program could do more to orient its workers in ways that prioritize family planning information and services in the context of primary health care service delivery. In particular, successful strategies for community outreach and doorstep services during the Navrongo experiment merit review and appraisal for possible adaptation to the Northern Regional context. Where demand for contraception is both pervasive and constrained, taking services to women in their homes can offset barriers to their seeking care elsewhere.

Third, concerns about side effects are widespread and pronounced. Improving the provision of information about side effects and expanding options for method choice for women could allay fears and possibly curtail the spread of misconceptions. Since such concerns are expressed in conjunction with concerns about social constraints to use, public discussion and communication about family planning, myths and rumors, and facts about side-effects should be convened in ways that include men in the process of community dialog. Throughout the northern regions of Ghana, community gatherings termed “durbars” are traditional mechanisms for community consensus building and collective action. Family planning-focused durbars could address the need for basic information about methods, side-effects, and benefits of use.

Fourth, CHPS should also direct particular attention to the need for outreach to male social networks and introduce ways to routinely convene community durbars that build male consensus for and understanding of family planning service activities. Social access to services represents a more prominent concern than geographic access. Services and information that are limited to clinical care will fail to address the need for social engagement, male participation, and community-based consensus that family planning practice is normal, acceptable, and healthy.

Finally, at the primary level of healthcare that CHPS represents, health workers are positioned to enhance the current favorable perceptions of the youth in these communities with supportive information. Youth are typically more educated than their parents, more likely to promote healthy childbearing practices, and positioned to catalyze social change. But engaging the entire community as a social system, rather than limiting family planning engagement to individual clinical encounters, is critical to building understanding and consensus that offsets social constraints to family planning adoption and practice among couples of childbearing age.

## Data Availability

By agreement among partners to the CHPS+ initiative, de-identified qualitative data are available to institutions seeking access by written request to Prof. Ayaga A. Bawah of this publication. Requests should clarify the purpose of research and should specify relevant ethical review for the proposed research. Data acquired by this process are for scholarly purposes only and may not be sold or independently distributed.

## Abbreviations

CHO: Community health officer
CHPS: Community-based Health Planning and Services
CPR: Contraceptive prevalence rate
FGD: Focus group discussion
GEHIP: Ghana Essential Health Interventions Programme
SLD: System Learning District
TFR: Total fertility rate

## Footnotes

1 We tested design balance by conducting a regression time-series analysis of the legacy of family planning indicators reported by the routinely compiled Ghana Health Service data of the national monitoring system for the four respective sub-districts where these four communities are located (Ghana Health Service, 2017). Results not shown here—but are available upon request—showed that none of the estimated areal indicator effects were statistically significant, establishing definitive evidence of the balance of family planning conditions prior to the present investigation and attesting to the validity of comparing qualitative data on social responses across contextually matched study localities. As of the onset of our research, the geographic density of CHPS services had no effect on the volume of new acceptors or repeat user clientele served by the program.
